# Impacts of decentralization in health systems in the state of São Paulo, Brazil

**DOI:** 10.31744/einstein_journal/2021GS5914

**Published:** 2021-07-01

**Authors:** Daniel Okita Uehara, Pedro Lucas Rosa, Matheus Cardoso Moraes, Renato Cesar Sato

**Affiliations:** 1 Universidade Federal de São Paulo São PauloSP Brazil Universidade Federal de São Paulo, São Paulo, SP, Brazil.

**Keywords:** Health services accessibility, Health equity, Geographic locations, Health facility, Health care rationing

## Abstract

**Objective:**

To evaluate a p-median model for health care services accessibility based on decentralization and optimal allocation of Primary Health Care Units in the State of São Paulo, Brazil.

**Methods:**

Using geographical data of Primary Health Care Units located in the State of São Paulo, potential support and supply facility allocations were simulated by means of a random approach. Several constraints were then imposed on the system to simulate different scenarios. Results were assessed according to geographic disposition.

**Results:**

Using a fixed number of supply facilities, ten as a constraint, the p-median approach allocated three facilities near the state capital (the area with the highest concentration of Primary Health Care Units), while remaining facilities were spread throughout the west of the state. A second round of tests assessed the impact of fixed costs alone on optimization, ranging from 71 optimal locations with a fixed unit cost to six optimal locations at a cost 300-fold higher. This finding was relevant to decision-making, since it encompassed scenarios in which only the final number of facilities or only the budget was known. A third set of simulations contemplates an intermediate scenario.

**Conclusion:**

The p-median approach was capable of optimizing a wide range of scenarios with an average running time of less than 2 hours and 30 minutes while considering a large dataset of more than 4,000 locations. In spite of some shortcomings, such as estimation of Euclidean distances, the method is simple yet powerful enough to be considered a useful tool to assist decision makers in the distribution of resources, and facilities across large areas with high number of locations to be supplied.

## INTRODUCTION

Decentralized health service networks offer the possibility of coverage to people living on the outskirts of cities, who would otherwise not have access to such services,^(1-5)^ and help to reduce overcrowding in large hospitals. However, the need to supply these infrastructures with consumables and medical equipment translates into higher costs. The consequences of bad decisions regarding allocation of new facilities extend far beyond higher costs, and may include higher mortality and morbidity rates.^(6-8)^ Such impacts are long lasting,^(9,10)^ since they are costly and difficult to revert. Deliberate decisions regarding the location of healthcare facilities should be based on prior comprehensive analysis of the scenario in question.

Cirino et al.,^(11)^ compared the actual allocation of hospitals in Santa Catarina with a simulation created using the p-median model to assist in decision-making regarding the construction of new facilities in regions with limited coverage. The p-median model may also be used to solve different problems in several research areas, such as the distribution of applicants according to optimal university entrance exam score,^(12,13)^ data clustering^(14,15)^ and several other studies, as shown by Farahani et al.,^(16,17)^ and Karatas et al.^(18)^

The expansion of decentralized structures results in a healthcare network that may contain thousands of facilities to be supplied and integrated across a vast area. When combined with factors such as deployment costs, selection of optimized spaces becomes a mathematical question. The solution to this problem is usually beyond the scope of the decision maker, who does not have the necessary tools to solve it. Development of novel optimization tools that can be run on standard personal computers introduces a new approach to the problem, allowing for a more reliable solution and the possibility of dealing with large numbers of constraints and data.

Decentralized facilities may provide services (storage and distribution of resources) to health care facilities. This research study was based on Primary Health Care Units, a social infrastructure project designed by the Brazilian Government to expand access to health care through the provision of free services of different medical specialties. In this study, some limitations, such as the streamlining of resource distribution to health units and improvement of quality of services for enhanced delivery of care to the population were accounted for.

## OBJECTIVE

To evaluate a p-median model for health care services accessibility based on decentralization and optimal allocation of Primary Health Care Units in the State of São Paulo, Brazil.

## METHODS

The model described in this article was implemented on a notebook with the following configurations: sixth generation Intel^®^ Core™ i5 processor, 8 GB RAM and Windows 10 operating system. The algorithm was implemented in Python 3.7.1 programming language using Jupyter Notebook as programming environment, the Anaconda Navigator software package and Gurobi™ version 8.1.1 (license acquired for academic purposes).^(19)^ This study was conducted at *Instituto de Ciência e Tecnologia* of *Universidade Federal de São Paulo* (UNIFESP).

The purpose of this research study was to create a model that would account for constraints and costs. The location of every Primary Health Care Units in the State of São Paulo determined according to latitude and longitude coordinates was used to generate a synthetic dataset including all possible facility locations. Finally, the model was optimized using a p-median approach for different constraint scenarios and results compared.

Given the approach selected, the project was exempt from ethical review. To optimize the distribution of facilities, the mathematical model considered the cost of selecting a given location, since the longer the distance from the Primary Health Care Units, the higher the cost of transportation. The following definitions were used: *i* for the location of existing health care facilities; and *j* for potential locations for implementing new facilities. The distance between these two points could then be calculated. An Euclidean approach^(20)^ is represented by the equation 1.

$$d_{ij}=\sqrt{\left(\left|x_{i}^{2}\right|-\left|x_{j}^{2}\right|\right)+\left(\left|y_{i}^{2}\right|-\left|y_{j}^{2}\right|\right)}$$ Equation 1

The distance d_ij_ between the Primary Health Care Units *i* and facility *j* can be associated with transportation expenses to incorporate the number of deliveries over time. Hence, the variable cost (v_ij_) can be deemed to be in direct proportion to transportation costs and distances involved, and estimated by using the function α, as equation 2.

$$v_{ij}=\alpha \cdot d_{ij}$$ Equation 2

Transportation-related costs were defined using equation 2. To calculate the total cost, the number of times each Primary Health Care Units uses the distribution facility must be considered, given the demand may vary between locations. Total cost was defined as y_ij_; and the cost associated with the fixed cost and implementation of each facility as f_j_. A binary variable x_ij_, usually known as p parameter, was used to represent the best allocations by determining that optimal location equals one and non-optimal location equals zero. In that manner, the desired total number of optimized facilities can be controlled. Hence, the total cost is described by the following equation 3.

$$C=\sum_{j\in J}f_{j}\cdot x_{j}+\sum_{j\in J}\sum_{i\in I}v_{ij}\cdot y_{ij}$$ Equation 3

To reduce the size of the dataset and consequently response time during the model optimization process, data in this study were limited to Primary Health Care Units located in the State of São Paulo. The dataset employed consisted of a database containing the geographic coordinates (*i.e.,* latitude and longitude) of 4,771 Primary Health Care Units listed on the register database on the last update.^(21)^

As regards potential locations for facilities, the best alternative to advance the project was to create simulations using random data. Simulations generated data with Gaussian distribution to ensure a more significant number of potential facility allocations.

Finally, using the abovementioned datasets, the model was optimized and compared under the following scenarios: data generated without and with outlier correction; using the p parameter as the only constraint; using costs as the only constraints. The map shows the optimized locations and their results.

## RESULTS

On the first test, the variable f_j_, which represented the fixed cost associated with implementing a facility at location *j*, was defined as a unit value for all possible locations. The variable representing the cost of transportation between the Primary Health Care Units and a given facility was also defined as a unit value and the variable p adjusted to ten (*i.e*., the optimization process was limited to the top ten points). The aim was to determine the optimal allocation of facilities based on minimal distances, without accounting for financial factors.

**Figure 1:**
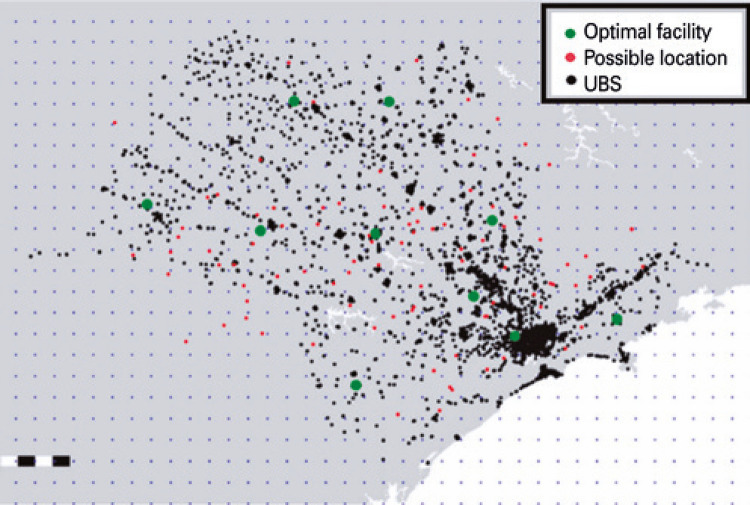
Optimal facility allocations considering p=10

Optimal locations determined by the model and respective latitude and longitude data were plotted on a base map as green dots (Figure 1). The optimization process lasted about 2 hours and 9 minutes.

UBS: *Unidade Básica de Saúde*.

In this study, three out of ten optimal locations determined were close to the state capital city, the area with the highest concentration of Primary Health Care Units in the state. Remaining units were sparsely distributed far away from the capital city, emphasizing the complexity of this type of task.

**Figure 2:**
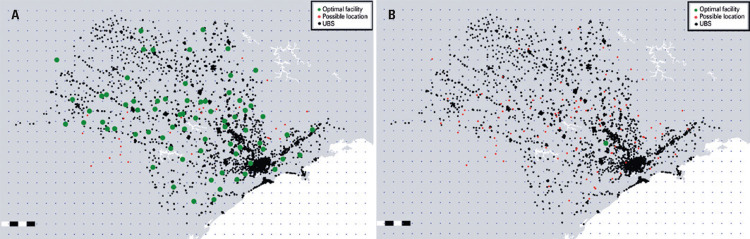
Optimal facility allocations: (A) Without the constraint imposed by the variable p; (B) Considering p=1

Given the purpose was to minimize distances between Primary Health Care Units and facilities, a second simulation disregarding the variable p, which in the previous model represented the constraint that limited the maximum number of facilities to be allocated, was created. Thus, using the fixed cost f_j_ as a unit value regardless of location, the process, which took about 2 hours and 11 minutes to be completed, returned a variable containing a set of 71 optimal locations for the model, expressed in figure 2A.

UBS: *Unidade Básica de Saúde*.

Results obtained underscore the importance that attributing a value to the variable p had in the model excluding implementation costs. Based on this principle, a third simulation was created, again attributing a value to variable p. This time, the value attributed corresponded to p=1, so that only the optimal location for implementing one facility to serve the entire state of São Paulo was determined. The result of this simulation, with a processing time of 2 hours and 31 minutes, can be seen in figure 2B.

In figure 2B, a green point could be seen near the metropolitan region of Campinas, the second largest city in the State of São Paulo. Given the state geography, the location of this facility appeared to meet the goal of minimizing average distances between Primary Health Care Units and facility. The proximity to the state capital (about 100km away), where the concentration of Primary Health Care Units was highest, was taken into account, and it represented the minimization of distances relative to Primary Health Care Units located in the west of the state.

**Figure 3:**
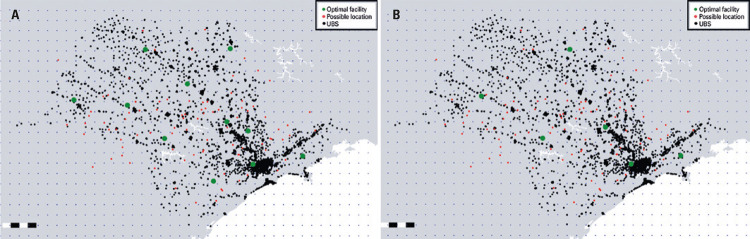
Optimal allocations with a fixed cost of (A) fj = 100 and (B) fj = 300

Controlling the value of the fixed costs associated with implementation of a facility at that location, and using values 100-fold or 300-fold higher than the one used in previous simulations, further simulations were created. These simulation processes took 2 hours and 11 minutes and 2 hours and 20 minutes to be completed, respectively, and results obtained are shown in figure 3.

UBS: *Unidade Básica de Saúde*.

Results derived from the latter simulation showed that the attribution of a cost f_j_*per se* also acted as constraining factor in the model, since it limited the number of facilities in the optimization process shown in figure 3, from 11 to six. This finding was of great significance for decision making, because it may provide decision makers with relevant information regarding the available budget for and the cost of each facility. In such conditions, the model must be able to keep minimizing costs involved. An intermediate scenario in which the number of facilities and the fixed cost were known may also be simulated using the method proposed, as both constraining factors can be simultaneously set.

Hence, three decision-making scenarios could be defined. In the first, the decision maker started from a limited budget regarding the number of facilities, and the model would interpret this based on the p parameter, with the allocation of the optimal number (“n desired”) of units. In the second, only the fixed cost of each space was known, and the model would interpret this as the fixed cost variable f_j_, and consider only the maximum number of facilities operating at that cost. In this manner, the cost of the initial investment in services may be offset by long-term savings obtained by reducing variable costs over time. The third scenario was an intermediate situation in which the maximum number of facilities was known and consequently expenses could be estimated.

## DISCUSSION

Delivery of consists of low-cost prevention strategies aimed to reach a large portion of the population.^(22)^ The growing number of studies investigating health care facility location gave rise to important collaborations between professionals to tackle this question.^(22,23)^

The wide array of constraining factors accounted for in the evaluation of methods based on p-median optimization demonstrates the flexibility of the model, which has also been emphasized in other research areas.^(24,25)^ Simulation running time was around 2 hours and 20 minutes on a standard notebook and considering a significant amount of locations. São Paulo is the most populated state in Brazil and has the largest number of Primary Health Care Units. This is thought to be an acceptable running time, given that allocations are not usually selected in 1 day, but rather after some days of analysis.

It should be also mentioned that potential locations had to be simulated in this study, which is not always the case in real-life applications. The coordinates of potential locations were accounted for in scenarios simulated in the model, reducing the effects of random input data on system dynamics.^(26)^

The supply facility considered in this study does not limit model generalization of this problem to health care. The distribution of sensitive medical equipment, such as positron emission tomography (PET) or magnetic resonance imaging (MRI) devices, which are available for general use at few units due to high cost,^(23,27)^ may also be modelled.

Moreover, the many factors that may impact the costs of implementing a facility at a given location were not accounted for in this study. Some examples of such factors include economic (local availability of qualified personnel and competition for such workers with other companies), political (tax incentives aimed to attract new companies) and environmental issues. All of these factors may constitute parameters to be included in the algorithm in future studies.

Minimization of average distances between demand (in this case, Primary Health Care Units) and facilities was also considered in the model. However, the model used the minimization of the Euclidean distance between points without accounting for the lack of different routes. Between every two points, an optimal Euclidean distance was obtained, which can be divergent due to the lack of a connecting highway.

## CONCLUSION

The study enabled the development of a simulation model to assist in decision-making process regarding the allocation of facilities, herein defined as units aimed to deliver services to Primary Health Care Units in the State of São Paulo. This research study attempted to show the importance of appropriate allocation of health care facilities with direct impacts on the quality of life of the population.

Results derived from implemented algorithms suggest the method proposed offers satisfactory solutions to the problem of health facility allocation at a low computational cost. Implementation of geospatial visualization by using the libraries available in the Python programming language was also vital to facilitate interpretation and analysis of findings.

However, the lack of a real set of possible facility locations limited simulation results and precluded the estimation of the applicability of such a model. Given the implemented algorithm and the possibility of geospatial visualization of the different simulations, the model proposed was thought to be a useful tool for health system-related decision-making process. However, the algorithm must be adjusted according to input variables, and potential limitations detected in previous simulations.

Further studies are warranted to address the shortcomings of the model, including use of new datasets, model adjustment to account for distance minimization and existing routes between points (*i.e*., not only Euclidean distances), and assessment of the demands of each facility according to size and service capacity.
