# Supplementary material for: Impacts of decentralization in health systems in the state of São Paulo, Brazil
Source: Einstein (Sao Paulo). 2021 Jul 1;19:eGS5914. doi: 10.31744/einstein_journal/2021GS5914 (PMC8253590; doi:10.31744/einstein_journal/2021GS5914)
Supplement: Translation

# Translation: Impactos da descentralização nos sistemas de saúde no estado de São Paulo, Brasil

**Authors:** Daniel Okita Uehara, Pedro Lucas Rosa, Matheus Cardoso Moraes, Renato Cesar Sato

**Affiliations:** 1 Brasil Universidade Federal de São Paulo, São Paulo, SP, Brasil.

**Keywords:** Acesso aos serviços de saúde, Equidade em Saúde, Localizações geográficas, Instalações de saúde, Alocação de recursos para a atenção à saúde

## Abstract

**Objetivo:**

Avaliar um modelo de p-medianas para acessibilidade de serviços de saúde com base na descentralização e na alocação ideal de Unidades Básicas de Saúde no estado de São Paulo, Brasil.

**Métodos:**

Por meio de dados geográficos das Unidades Básica de Saúde do estado de São Paulo, possíveis alocações de suporte e suprimento foram simuladas em uma abordagem randomizada. Em seguida, uma série de restrições foi imposta ao sistema, simulando possíveis cenários, e os resultados foram verificados por disposição geográfica.

**Resultados:**

Dado um número de instalações desejadas, dez como restrição, a abordagem p-mediana alocou três nas proximidades da capital, área de maior concertação de Unidades Básica de Saúde, e as demais foram distribuídas a oeste. Uma segunda rodada de testes avaliou exclusivamente a influência dos custos fixos, variando de 71 posições ótimas, com custo fixo unitário, até seis unidades, para um custo 300 vezes maior. Isso foi relevante no processo de tomada de decisão, pois abrangeu cenários em que apenas o número de instalações finais era conhecido e em que apenas o orçamento estava especificado. Um terceiro conjunto de simulações contemplou uma mistura dos casos anteriores.

**Conclusão:**

A abordagem p-mediana foi capaz de otimizar uma ampla gama de cenários, com média de tempo de execução abaixo de 2 horas e 30 minutos, considerando um conjunto de dados com mais de 4.000 posições. Apesar de suas deficiências, considerando distâncias euclidianas, o método é simples, mas poderoso para ser considerado como uma ferramenta para o tomador de decisão em relação à distribuição de recursos e de instalações em grandes áreas e alto número de locais considerados.

## INTRODUÇÃO

Redes descentralizadas de serviços de saúde abrem a possibilidade de cobertura para indivíduos que vivem na periferia das cidades e que, de outra forma, não poderiam acessá-las,^(1-5)^ além de ajudar a reduzir a superlotação em grandes hospitais. Entretanto, a necessidade de abastecer essas infraestruturas com bens de consumo e equipamentos médicos se traduz em custos mais elevados. As consequências de decisões equivocadas quanto à alocação de novas unidades vão muito além de custos elevados, podendo incluir taxas mais altas de mortalidade e morbidade.^(6-8)^ Esses impactos são duradouros,^(9,10)^ por serem onerosos e difíceis de reverter. Decisões deliberadas quanto à localização de unidades de saúde devem ser baseadas em análises abrangentes prévias do cenário em questão.

Cirino et al.,^(11)^ compararam a real alocação de hospitais em Santa Catarina com uma simulação feita empregando-se o modelo de p-medianas para auxiliar na tomada de decisão quanto à construção de novas unidades em regiões com cobertura limitada. O modelo de p-medianas também pode ser empregado na solução de diversos problemas em várias áreas de pesquisa, como a distribuição de candidatos de acordo com a pontuação ideal no vestibular,^(12,13)^ o agrupamento de dados^(14,15)^ e diversos outros estudos, conforme demonstrado por Farahani et al.,^(16,17)^ e Karatas et al.^(18)^

A expansão de estruturas descentralizadas resulta em uma rede de cuidados de saúde que pode conter milhares de unidades a serem abastecidas e integradas em uma grande área. Quando combinada com fatores, como custos de implementação, a seleção de espaços otimizados se torna uma questão matemática. A solução desse problema, em geral, está fora da alçada do responsável pela tomada de decisão, que não possui as ferramentas necessárias para solucioná-lo. O desenvolvimento de novas ferramentas de otimização que possam ser executadas em computadores pessoais padrão introduz uma nova abordagem do problema, permitindo a obtenção de uma solução mais confiável e possibilitando lidar com um grande número de limitações e dados.

Unidades descentralizadas podem prestar serviços (armazenagem e distribuição de recursos) para unidades de saúde. Esta pesquisa foi baseada em Unidades Básicas de Saúde (UBS), um projeto de infraestrutura social criado pelo governo brasileiro para expandir o acesso aos cuidados de saúde por meio da prestação de serviços gratuitos em diversas especialidades médicas. Neste estudo, foram consideradas limitações como o alinhamento da distribuição de recursos para unidades de saúde e a melhoria da qualidade dos serviços para oferecer cuidados de saúde melhores para a população.

## OBJETIVO

Avaliar um modelo de p-medianas para acessibilidade de serviços de saúde com base na descentralização e na alocação ideal de Unidades Básicas de Saúde no estado de São Paulo, Brasil.

## MÉTODOS

O modelo descrito neste artigo foi implementado em um *notebook* com as seguintes configurações: processador Intel^®^ Core™ i5 de sexta geração, 8 GB de RAM e sistema operacional Windows 10. O algoritmo foi implementado na linguagem de programação Python 3.7.1, empregando-se o Jupyter Notebook como ambiente de programação e os programas Anaconda Navigator e Gurobi™ versão 8.1.1 (licença para fins acadêmicos).^(19)^ Este estudo foi realizado no Instituto de Ciência e Tecnologia da Universidade Federal de São Paulo (UNIFESP).

O objetivo deste estudo foi criar um modelo que levasse em conta as limitações e os custos. A localização de cada UBS do estado de São Paulo determinada de acordo com as coordenadas de latitude e longitude foi utilizada para gerar um conjunto de dados sintético, incluindo todas as possíveis localizações de unidades. Finalmente, o modelo foi otimizado por meio de uma abordagem de p-medianas para diferentes cenários de limitações, e os resultados foram comparados.

Dada a abordagem selecionada, o projeto foi isento de revisão ética. A fim de otimizar a distribuição das unidades, o modelo matemático levou em conta os custos da seleção de uma determinada localização, pois quanto maior a distância da UBS, maior o custo do transporte. Foram empregadas as seguintes definições: *i* para localização das unidades de saúde existentes e *j* para possíveis localizações para instalação de novas unidades. A distância entre esses dois pontos pode, então, ser calculada. Uma abordagem euclidiana^(20)^ foi representada pela equação 1.

$$d_{ij}=\sqrt{\left(\left|x_{i}^{2}\right|-\left|x_{j}^{2}\right|\right)+\left(\left|y_{i}^{2}\right|-\left|y_{j}^{2}\right|\right)}$$ Equação 1

A distância d_ij_ entre a UBS *i* e a unidade *j* pode ser associada aos gastos com transporte a fim de incorporar o número de entregas ao longo do tempo. Dessa forma, a variável custo (v_ij_) pode ser considerada diretamente proporcional aos custos do transporte e às distâncias envolvidas, sendo estimada empregando-se a função α, conforme a equação 2.

$$v_{ij}=\alpha \cdot d_{ij}$$ Equação 2

Os custos gerados pelo transporte foram definidos conforme a equação 2. Para calcular o custo total, o número de vezes que cada UBS usa a unidade de distribuição deve ser considerado, uma vez que a demanda pode variar entre as localidades. O custo total foi definido como y_ij_, e o custo associado ao custo fixo e à instalação de cada unidade foi definido como f_j_. Uma variável binária x_ij_, geralmente conhecida como parâmetro p, foi usada para representar as melhores alocações, convencionando-se que a localização ideal é igual a um e a localização não ideal é igual a zero. Dessa forma, o número total desejado de unidades otimizadas pode ser controlado. Assim, o custo total é descrito pela equação 3.

$$c=\sum_{j\in J}f_{j}\cdot x_{j}+\sum_{j\in J}\sum_{i\in I}v_{ij}\cdot y_{ij}$$ Equação 3

A fim de diminuir o tamanho do conjunto de dados e, consequentemente, o tempo de resposta durante o processo de otimização do modelo, os dados empregados neste estudo foram limitados ao estado de São Paulo. O conjunto de dados consistiu em um banco de dados que continha as coordenadas geográficas (latitude e longitude) de 4.771 UBS listadas no registro na data da última atualização.^(21)^

No que se refere à possível alocação de unidades, a melhor alternativa para dar continuidade ao projeto foi criar simulações com dados aleatórios. As simulações geraram dados com distribuição gaussiana para garantir um número mais significativo de possíveis alocações de unidades.

Finalmente, usando-se os conjuntos de dados descritos, o modelo foi otimizado e comparado nas seguintes situações: dados gerados sem e com correção dos discrepantes (*outliers*), empregando-se o parâmetro p como única limitação e utilizando os custos como única limitação. O mapa mostra a localização otimizada e seus resultados.

## RESULTADOS

No primeiro teste, a variável f_j_, que representava o custo fixo associado à implementação de uma unidade na localidade *j*, foi definida como um valor unitário para todas as localidades possíveis. A variável que representava o custo do transporte entre a UBS e uma unidade também foi definida como um valor unitário, enquanto a variável p foi ajustada para dez (isto é, o processo de otimização foi limitado aos dez pontos mais altos). O objetivo foi determinar a alocação ideal das unidades com base nas distâncias mínimas, sem considerar os fatores financeiros.

As localizações ideais determinadas pelo modelo e as respectivas latitude e longitude foram plotadas em um mapa base como pontos verdes (Figura 1). O processo de otimização levou em torno de 2 horas e 9 minutos.

**Figura 1:** Alocação ideal de unidades considerando-se p=10

UBS: Unidade Básica de Saúde.

Neste estudo, três das dez localizações ideais encontradas ficavam próximas da capital do estado, que era a área com a maior concentração de UBS do estado. As unidades restantes encontravam-se esparsamente distribuídas em áreas distantes da capital, enfatizando a complexidade desse tipo de tarefa.

Uma vez que o objetivo era minimizar as distâncias entre as UBS e as unidades, foi criada uma segunda simulação, desconsiderando a variável p, que, no modelo anterior, representava a limitação que restringia o número máximo de unidades a serem alocadas. Assim, empregando-se o custo fixo f_j_ como valor unitário independentemente da localização, o processo, que levou em torno de 2 horas e 11 minutos para ser completado, retornou uma variável que continha um conjunto de 71 localidades ideais para o modelo, o qual foi expresso na figura 2A.

**Figura 2:** Alocações ideais de unidades: (A) Sem a limitação imposta pela variável p; (B) Considerando-se p=1

UBS: Unidade Básica de Saúde.

Os resultados obtidos reforçaram a importância que a atribuição de um valor para a variável p teve no modelo que excluiu os custos de instalação. Com base nesse princípio, uma terceira simulação foi realizada, mais uma vez atribuindo um valor à variável p. Desta vez, o valor atribuído foi p=1, de tal forma que apenas a localização ideal para instalação de uma unidade que servisse todo o estado de São Paulo fosse determinada. O resultado dessa simulação, cujo tempo de processamento foi de 2 horas e 31 minutos, pode ser visualizado na figura 2B.

Na figura 2B, um ponto verde pode ser visualizado na Região Metropolitana de Campinas, a segunda maior cidade do estado de São Paulo. Dada a geografia do estado, a localização dessa unidade, inicialmente, satisfez o objetivo de minimizar as distâncias médias entre a UBS e a unidade. A proximidade da capital do estado (aproximadamente 100km de distância), onde a concentração de UBS era mais alta, foi considerada e representou a minimização das distâncias relativas às UBS localizadas no oeste do estado.

Controlando-se o valor dos custos fixos associados à instalação de uma unidade naquela localidade e empregando-se valores 100 ou 300 vezes mais altos do que o empregado nas simulações anteriores, novas simulações foram geradas. Esses processos de simulação levaram 2 horas e 11 minutos e 2 horas e 20 minutos para serem completados, respectivamente, e os resultados obtidos podem ser visualizados na figura 3.

**Figura 3:** Alocações ideais com custo fixo de (A) fj. = 100 e (B) fj = 300

UBS: Unidade Básica de Saúde.

Os resultados da última simulação mostraram que a atribuição de um custo f_j_ por si só também agiu como fator limitante no modelo, uma vez que restringiu o número de unidades no processo de otimização mostrado na figura 3, de 11 para seis. Esse achado se revestiu de grande importância na tomada de decisão, pois pôde fornecer informações relevantes aos tomadores de decisão quanto ao orçamento disponível e ao custo de cada unidade. Nessas condições, o modelo pôde ser capaz de continuar a minimizar os custos envolvidos. Uma situação intermediária, em que o número de unidades e o custo fixo fossem conhecidos, também poderia ser simulada empregando-se o método proposto, uma vez que ambos os fatores limitantes poderiam ser determinados simultaneamente.

Dessa forma, três cenários de tomada de decisão puderam ser definidos. No primeiro, o responsável pela tomada de decisão partiu de um orçamento limitado quanto ao número de unidades, e o modelo interpretou isso com base no parâmetro p, com alocação do número ideal (“n desejado”) de unidades. No segundo, somente o custo fixo de cada espaço foi conhecido, e o modelo interpretou isso como a variável custo f_j_, considerando apenas o número máximo de unidades que operaram com esse custo. Dessa forma, o custo do investimento inicial em serviços pôde ser compensado pela economia gerada a longo prazo, por meio da redução dos custos variáveis ao longo do tempo. O terceiro cenário correspondeu a uma situação intermediária em que o número máximo de unidades era conhecido e, consequentemente, as despesas podiam ser estimadas.

## DISCUSSÃO

A prestação de cuidados de saúde consiste em estratégias preventivas de baixo custo voltadas para uma grande proporção da população.^(22)^ O número crescente de estudos investigando a localização otimizada de unidades de saúde deu origem a colaborações importantes entre os profissionais na abordagem dessa questão.^(22,23)^

A ampla gama de fatores limitantes considerados na avaliação de métodos baseados em otimização de p-medianas demonstra a flexibilidade do modelo, que também foi destacada em outras áreas de pesquisa.^(24,25)^ O tempo necessário para execução das simulações em um *notebook* padrão, levando em conta uma quantidade considerável de localidades, foi de, aproximadamente, 2 horas e 20 minutos. São Paulo é o estado mais populoso e com maior número de UBS do Brasil. O tempo de execução em questão foi considerado aceitável, uma vez que, via de regra, as alocações não são selecionadas em 1 dia, e sim depois de dias de análise.

Outro ponto importante foi a necessidade de simulação de possíveis localizações neste estudo, o que nem sempre ocorre em aplicações reais. As coordenadas das possíveis localizações foram consideradas nas situações simuladas no modelo, reduzindo o efeito da introdução de dados aleatórios sobre a dinâmica do sistema.^(26)^

A unidade de abastecimento considerada neste estudo não restringe a generalização do modelo desse problema aos cuidados de saúde. A distribuição de equipamentos médicos sensíveis, como aparelhos para tomografia por emissão de pósitrons (PET) e ressonância magnética (RM), disponíveis para uso geral em poucas unidades, devido ao seu alto custo,^(23,27)^também pode ser modelada.

Além disso, os inúmeros fatores que podem influenciar nos custos da instalação de uma unidade em um determinado local não foram considerados neste estudo. Exemplos de tais fatores incluem questões econômicas (disponibilidade local de pessoal qualificado e concorrência por esse tipo de mão de obra com outras empresas), políticas (incentivos fiscais destinados a atrair novas empresas) e ambientais. Todos esses fatores constituem parâmetros a serem incluídos no algoritmo em estudos futuros.

A minimização das distâncias médias entre a demanda (no caso, a UBS) e as unidades também foi levada em conta no modelo. Entretanto, o modelo empregou a minimização da distância euclidiana entre os pontos, sem considerar a ausência de rotas diferentes. Entre cada dois pontos, foi obtida uma distância euclidiana ideal, que pode ser divergente, dada a falta de uma via direta de conexão.

## CONCLUSÃO

Este estudo permitiu o desenvolvimento de um modelo de simulação para auxiliar na tomada de decisão referente à alocação de instalações, aqui definidas como unidades de prestação de serviços para Unidades Básicas de Saúde no estado de São Paulo. Este trabalho de pesquisa tentou mostrar a importância da alocação adequada de unidades de saúde, a qual apresenta impacto direto na qualidade de vida da população.

Os resultados obtidos por meio dos algoritmos implementados sugerem que o método proposto oferece soluções satisfatórias para o problema da alocação de unidades de saúde a um baixo custo computacional. A implementação da visualização geoespacial possibilitada pelo uso das bibliotecas da linguagem de programação Python também foi fundamental para a compreensão e a análise dos achados.

Entretanto, a falta de um conjunto real de possíveis localizações de unidades limitou os resultados da simulação e impediu a estimativa da aplicabilidade do modelo. Considerando-se a implementação do algoritmo e a possibilidade de visualização geoespacial das diferentes simulações realizadas, o modelo proposto foi considerado uma ferramenta útil para o processo de tomada de decisões relacionadas ao sistema de saúde. Ainda assim, o algoritmo precisa ser ajustado de acordo com as variáveis consideradas e as potenciais limitações detectadas em simulações anteriores.

Novos estudos se fazem necessários para abordar a deficiências do modelo, incluindo o uso de novos conjuntos de dados, o ajuste do modelo considerando a minimização das distâncias e as rotas existentes entre os pontos (ou seja, a distância não euclidiana) e a avaliação das demandas de cada unidade, de acordo com o respectivo tamanho e a capacidade de serviço.
